# The Cost of Lunacy

**Published:** 1906-06-23

**Authors:** H. R. Gawen Gogay

**Affiliations:** ex-Guardian of St. Saviour's Union; Gawenhurst, Southchurch Beach, Essex


### Editor's Letter-Box.

### THE COST OF LUNACY.

Mr. H. R. Gawen Gogay, ex-Guardian of St. Saviour's Union, writes to us from Gawenhurst, Southchurch Beach, Essex : Will you kindly permit me to draw the attention of the ratepayers in England and Wales to the following extraordinary figures dealing with certified pauper lunacy, taken from the Thirty-fourth Annual Report of the Local Government Board, pages 460 and 461? From 1880 to 1904, a period of twenty-five years, I find the nation has spent no less a sum than £36,264,702 sterling on pauper lunacy. The report only carries us to 1904, but if we assume the cost for 1905 and 1906 to be the same as 1904—a very moderate computation, seeing that since 1880 the cost has been regularly increasing annually—we get a total to date (a period of twenty-seven years) of over £41,0C0,000 sterling as the cost of pauper lunacy ! Sir, there must be something radically wrong somewhere; this is self-evident, and needs no demonstration. On page 445 of the same report we find the number of lunatics has jumped from 65,345 in 1881 to 109,100 in 1905. A more recent return puts the number to date at 120,000 souls afflicted with the scourge of insanity ! Thanking you in anticipation.

. ·. The subject of the above letter is one which has often been referred to in these columns. The remedy is by no means simple, as many issues are involved ; but the following are among the most salient points for reformers, and they may be divided into two heads : First, the reduction in the number of the insane for the future by curtailing the use of any form of alcohol, and the prohibition of marriage in people tainted with hereditary insanity. These two causes account for about 50 per cent. of insanity. Second, the more economical maintenance of the existing number of lunatics. This could be easily carried out by the retention or admission in the lunatic wards of our workhouses of those patients who are merely imbecile or idiotic; and this could be effected by diverting the capitation grant from the asylum to the workhouse. Our recent county asylums are not only unnecessarily extravagant in their building, but they are being for ever added to; and the cost has gone up from about £100 a bed to £300 to £400. Large sums are spent in such things as pathological departments and research, which, however desirable in themselves, are no part of the duty of committees. It is not to the curing of an additional 1 per cent. of cases that we must look for the lightening of the burden mentioned by our correspondent, but by the prevention of the disease. A reconstitution or a reform in the Board of Lunacy on the lines advocated lately in the House of Commons might be tried. If our correspondent would devote the rest of his life to dissecting the whole complex question, and could inculcate his views on the people, he would be a great benefactor to his age and country.—Editor, The Hospital.

